# Sonographic Differential Diagnosis in Deep Infiltrating Endometriosis: The Bowel

**DOI:** 10.1155/2019/5958402

**Published:** 2019-10-28

**Authors:** Marco Scioscia, Simone Orlandi, Giamberto Trivella, Antonella Portuese, Stefano Bettocchi, Giovanni Pontrelli, Paolo Bocus, Bruna Anna Virgilio

**Affiliations:** ^1^Department of Obstetrics and Gynecology, Policlinico Hospital, Abano Terme, Padua, Italy; ^2^Department of Obstetrics and Gynecology, Sacro Cuore Don Calabria Hospital, Negrar, Verona, Italy; ^3^Department of Gastroenterology and Endoscopy, Sacro Cuore Don Calabria Hospital, Negrar, Verona, Italy; ^4^Progetto Salute, Radiology Diagnostic Center, Verona, Italy; ^5^Unit of Obstetrics and Gynecology, Department of Biomedical and Human Oncologic Science, Policlinico University of Bari, Bari, Italy

## Abstract

Up to one-third of fertile-age women with severe endometriosis suffer from colonic involvement. Transvaginal ultrasonography has become a first-line diagnostic tool for the study of the pelvis and more specifically for the diagnosis of pelvic endometriosis. Accuracy of pelvic ultrasound for deep endometriosis increases with operator experience, but the difficulties in the differential diagnosis with diseases that can afflict the bowel tract remain a challenge. We reviewed noteworthy cases referred for secondary level diagnosis suspected of bowel endometriosis in which the subsequent ultrasound led to an alternative diagnosis. This case series aims to highlight awareness for both experts and less-experienced operators the possible differential diagnoses of bowel lesions that initially resemble endometriosis.

## 1. Introduction

Endometriosis is a common disease of reproductive-age women with an estimated prevalence between 5 and 15% [1, 2]. Certainly, clinical history, pelvic bimanual examination, and ultrasound represent the first-line methods to diagnose or to suspect endometriosis while a second-level ultrasound (ultrasound with an expert in the field) can improve the detection accuracy of most of the pelvic locations of this disease [3]. The great difficulty in complete sonographic evaluation of pelvic endometriosis is mostly related to the variance in the appearance of endometriosis lesions and the distorted anatomy secondary to adhesions and fibrosis. In fact, endometriosis induces an intense reactive sterile inflammation that lead to the formation of adhesions and reactive fibrosis [4]. Furthermore, endometriosis can extend to organs other than the genitalia including the bowel, the bladder, and retroperitoneal structures (ureters, parametria, and nerves) [5], thus confounding the ultrasound evaluation [6].

Preoperative diagnosis of intestinal endometriosis has proven a challenge. Colonic endometriosis that affects about 37% of women with severe endometriosis is not a rare condition of the disease [5]. Correct diagnosis is fundamental to formation of the appropriate treatment strategy [7]. In the recent years, the ultrasound detection rate of endometriosis foci of the bowel has increased (high sensitivity and specificity) [8, 9], with accuracy rates as high as other imaging techniques [10, 11]. The vast majority of studies evaluating ultrasound in bowel endometriosis are aimed at gauging the detection rate in all pelvic locations or a comparison of ultrasound with other imaging methods [10–12]. Comparison of data between the different published studies is difficult because of the heterogenicity in the terminology used to describe structures and anatomical locations. In this regard, a consensus was published to standardize methodology and nomenclature in ultrasound for endometriosis [13]. However, none of them emphasized the important aspect of differential diagnosis. There have been a limited number of studies describing the sonographic differential diagnosis in endometriosis focused on ovarian cysts only [8, 14]. The differential diagnosis of bowel lesions resembling endometriosis locations may in some cases prove challenging. This report aims to provide several practical suggestions for the sonographic differential diagnosis in endometriosis of cases with suspected bowel involvement.

## 2. Methods

This is a retrospective review of sonographic images and surgical records of endometriosis cases where the ultrasound preoperative diagnosis appeared difficult and posed interesting cues for differential diagnosis between the years 2015 and 2018. All patients were referred for suspected endometriosis to either of the two referral centers in Italy for this disease, the Sacro Cuore Don Calabria Hospital (Negrar, Italy) and the Policlinico Hospital (Abano Terme, Italy). Each center performs about 2,500 ultrasound scans for endometriosis per year. All cases reported underwent laparoscopy with surgical removal of the lesion to obtain a histological evaluation. All scans were performed by gynecologists (MS, GT, and BAV) with at least 10 years of experience in gynecological ultrasound with a special interest in endometriosis. Transrectal scans were carried out by expert gastroenterologists (SO and PB) experienced in bowel ultrasound. The ultrasound examiners had access to the patient's history, but they neither performed gynecological or rectal examination nor were aware of the suspect derived from other imaging techniques (MRI or CT scans). Transvaginal, transabdominal and, when required, transrectal ultrasound were performed. Transrectal ultrasound was used to differentiate extrinsic from endophytic (growing inward) lesions. A standardized ultrasound technique was used by gynecologists in all cases: (i) the bladder was not completely emptied to evaluate the internal bladder surface and to allow the evaluation of the cervix and vagina also transabdominally; (ii) particular attention was paid at the time of introduction of the transvaginal probe to explore (also by translabial ultrasound) the vaginal wall, perivaginal tissues, distal rectum, urethra, and recto- and vesico-vaginal septa; (iii) the examination was extended to all relevant organs (uterus, adnexa, bladder, rectum, distal sigmoid colon, and retroperitoneal structures); (iv) the “sliding sign” was always evaluated to assess the presence of adhesions or endometriosis noduli; (v) transabdominal ultrasound was always performed to obtain a second viewpoint of pelvic lesions, to evaluate large masses and to assess the presence of hydronephrosis (as an indirect sign of ureteral obstruction).

## 3. Results

**Figure 1:**
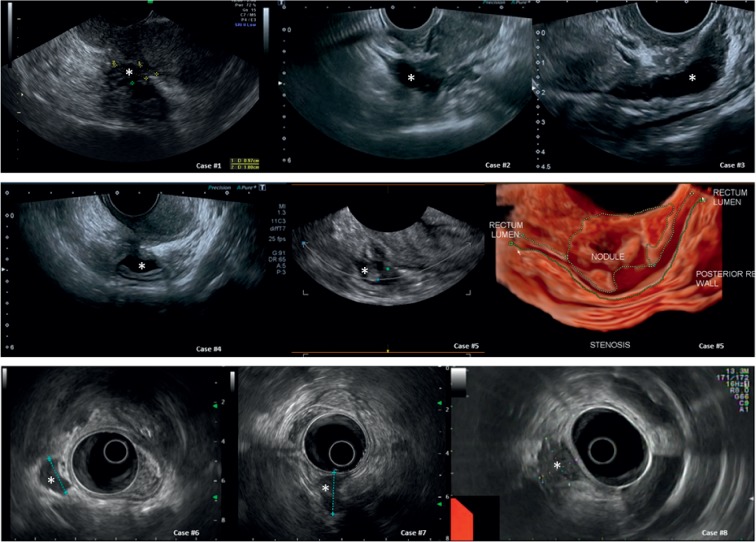
Images of bowel endometriosis (sonographic details of each case are reported in Table 1).

**Figure 2:**
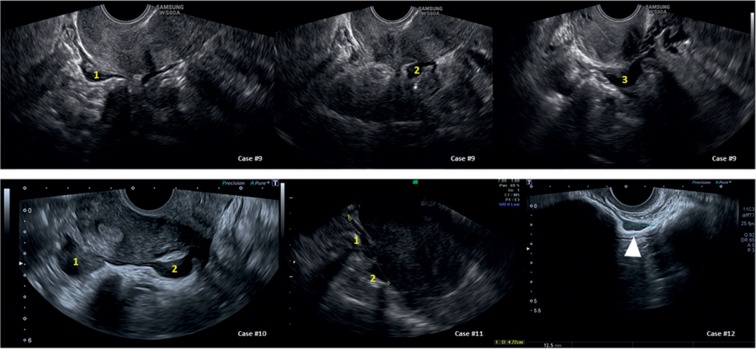
Differential diagnosis for bowel endometriosis (sonographic details of each case are reported in Table 2).

**Figure 3:**
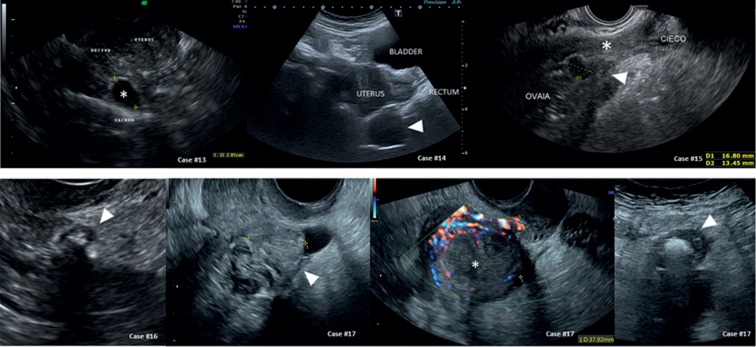
Images of multifocal bowel endometriosis (sonographic details of each case are reported in Table 3).

**Figure 4:**
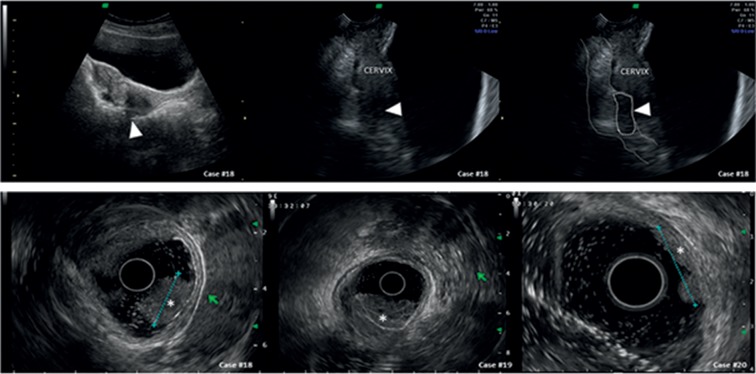
Differential diagnosis with bowel polyps and cancer (sonographic details of each case are reported in Table 4).

We selected twenty cases that were referred for secondary evaluation of endometriosis. The first 12 cases report endometriosis of the bowel as it appears in normal and less-frequent cases. For patients 13 to 20 (Figures 1 and 2, [Supplementary-material supplementary-material-1]), we report 8 cases referred with suspected endometriosis while another benign (Figure 3) or malignant (Figure 4) diagnosis was made. The histological diagnosis obtained after surgery is reported in the tables beneath the sonographic suspect as “confirmed diagnosis” if the pathological diagnosis was correspondent to the preoperatory ultrasound suspect or as “missed diagnosis” with the final histological evaluation.

All images are reported in matched figures and tables (from 1 to 4) so that all ultrasound details are shown in linked table. A video ([Supplementary-material supplementary-material-1]) is supplied showing the dynamic main ultrasound aspects of bowel endometriosis, appearance in a 3D evaluation, and the key differences in lesion growth for endometriosis, polyps, and cancer of the rectum.

Bowel lesions (Figure 1) can appear with different shapes, but an anechoic appearance without posterior enhancement is always found; they can encroach the bowel lumen, and their limits can be digitiform (see case #1), irregular (cases #2 and #3), or smooth (see #4 and #5). The “sliding sign” is very often negative as the nodule may involve the Douglas pouch and the uterosacral ligaments or can be attached to the posterior wall of the uterus.

Three-dimensional (3D) ultrasound has been proposed as a valuable tool to improve the diagnostic accuracy of 2D ultrasonography [15, 16] although conflicting results were reported [10]. Certainly, the definition of the 3D imaging can provide further details about the infiltration of the muscularis of the bowel and display lesion characteristics that outperform degree of stenosis (see case #5 in Figure 1 and [Supplementary-material supplementary-material-1], Samsung's “Crystal Vue™” Ultrasound Imaging Technology was used on Samsung WS80 Elite system, Samsung Medison Co. Ltd., Seoul, South Korea). In some cases, especially when the transvaginal ultrasound produces unclear imaging (i.e., large endometriomas may be attached to the bowel surface and can reduce the likelihood of detection of the lesion or to assess the extension and infiltration of the muscularis), rectal endoscopic ultrasound with a radial probe can be used (see cases #6 to #8 in Figure 1) [17].

Bowel endometriosis may present as a multifocal disease [18] (see cases #9 and #10, Figure 2), and this has to be differentiated with familial polyposis and rectal cancer. Women with endometriosis are usually younger than patients with colonic cancer, and family history is certainly of help in cases of bowel polyposis. Furthermore, rectal polyps (see #18 of Figure 4) and cancer (#19 and #20 of Figure 4) grow inside the lumen (advanced colonic cancer can infiltrate the bowel wall) while endometriosis starts from the serosa and encroaches the bowel from outside (see discussion). In the case #18 (Figure 4), a virgin woman underwent ultrasound for endometriosis suspect. A lesion of the bowel was seen, but it appeared as an intraluminal lesion of the bowel (arrow). A transrectal ultrasound was performed and supported the suspect of a rectal polyp diagnosed with a rectal endoscopic ultrasound with a radial probe and a subsequent colonic endoscopy. Rectal cancer can infiltrate the entire bowel wall in advanced stages (see case #19, Figure 4), but the presence of specific symptoms (rectal bleeding, unexplained weight loss, frequent gas pains, or stomach cramps) can lead to the correct diagnosis.

Small bowel involvement in endometriosis cases is a quite rare condition with an incidence between 0.5 and 4.7% [19]. The terminal ileum and the ileocecal valve are the most frequent locations although other ileal tracts can be affected. Preoperative sonographic diagnosis is usually not possible although it can be seen sporadically as in case #12 in Figure 2. The small bowel was seen in the vesico-uterine pouch and presented as a typical endometriosis lesion of the bowel (see cases #9 and #10 of the same figure). The differential diagnosis with a nodule of the peritoneum of the vesico-uterine pouch was made by observing the presence of peristalsis and positive “sliding sign” (confirmed at laparoscopy).

It is important to note for the differential diagnosis that rectum endometriosis always involves the anterior wall of the sigmoid rectum. Cases #13 and #14 (Figure 3) exhibit two cases referred for bowel endometriosis that displayed a cyst behind the rectum, just above the sacral surface, in asymptomatic patients. In case #13, the cyst was anechoic with well-defined borders and the posterior wall of the rectum appeared intact and smooth, so a Tarlov cyst was hypothesized (confirmed diagnosis at MRI scan). The other case (#14 of Figure 2) showed a well-defined round mass (arrow) with fine low-level echoes between the bowel and the sacrum; the cyst compressed and displaced the rectum without any sign of infiltration, and the Doppler evaluation was considered not satisfactory. According to the position (behind the rectum), the aspect (presacral endometriosis presents as a solid and irregular lesion, and it is usually very small and often undetectable at ultrasound), and the absence of symptoms (presacral endometriosis induces a pain that spreads out over one or both legs as a sciatalgic pain), a sacral ganglia disease was presumed (a ganglioneuroma was confirmed at histology).

Endometriosis of the appendix is a rare condition with a prevalence between 0.4 and 2.8% of reproductive-age women [20]. Its preoperative diagnosis is sporadic, but it should be differentiated from an appendicular abscess. In case #15 of Figure 3, we report a case of appendicular abscess with the caecum that was dislocated down into the pelvis, the appendix (^*∗*^) appeared thick and attached to the right ovary with an irregular hypoechoic area between the two organs (arrow). In such a case, abdominal pain with high inflammatory blood tests certainly suggest appendicitis regardless of whether it is superimposed to endometriosis. Endometriosis of the appendix is usually clinically silent or associated with chronic pelvic pain but not with sharp pain in the right lower abdominal quadrant, vomiting, abdominal swelling, and inability to pass gas as in appendicitis [20].

Occasionally, it can be difficult to distinguish bowel endometriosis from colonic diverticula. The main characteristics of diverticula are that they grow outwards and show a thick bowel wall with a hyperechoic content (see case #16 of Figure 3, arrow). The picture can be more complex if an inflammation of the diverticulum occurs with a close abscess (see case #17, Figure 3). In this case, an undefined oval mass was identified between the uterus and the sigmoid colon. A transverse section demonstrated the presence of a diverticulum (arrow) with an intense positivity at Doppler evaluation of the bowel wall. Clinical symptoms (constant abdominal tenderness, nausea and vomiting, and pyrexia) and inflammatory blood tests can support the sonographic hypothesis and can lead to the correct diagnosis.

## 4. Discussion

For many years, the main challenge in ultrasound for endometriosis has been the detection and accuracy of deep infiltrating endometriosis and the evaluation of the extension of the disease in nonovarian organs [3, 11, 13, 21–26]. Recently, Exacoustos et al. [9] published an excellent overview on the ability of ultrasound to identify endometriosis lesions not only demonstrating where and what to observe but also highlighting the role of the real-time dynamic ultrasound to improve the accuracy. Several articles have focused on the comparison between ultrasound and other imaging techniques [10, 12, 27], while very few dealt with a very complex subject like the differential diagnosis focusing on ovarian cysts and adenomyosis only [9, 14, 28].

This investigation reports cases where the differential diagnosis may be difficult as they present similar sonographic features. Sometimes, the differential diagnosis of deep infiltrating endometriosis of the bowel may not be easy. In fact, even experts in gynecological ultrasound can fail to correctly diagnose a disease as reported in the literature [29]. This highlights the difficulty involved in the ultrasound diagnosis of endometriosis can be.

Significant comments should be made about colonic endometriosis and cancer. The cases presented in this paper were reported as these patients were referred for a suspect of bowel endometriosis in women of reproductive age.

**Figure 5:**
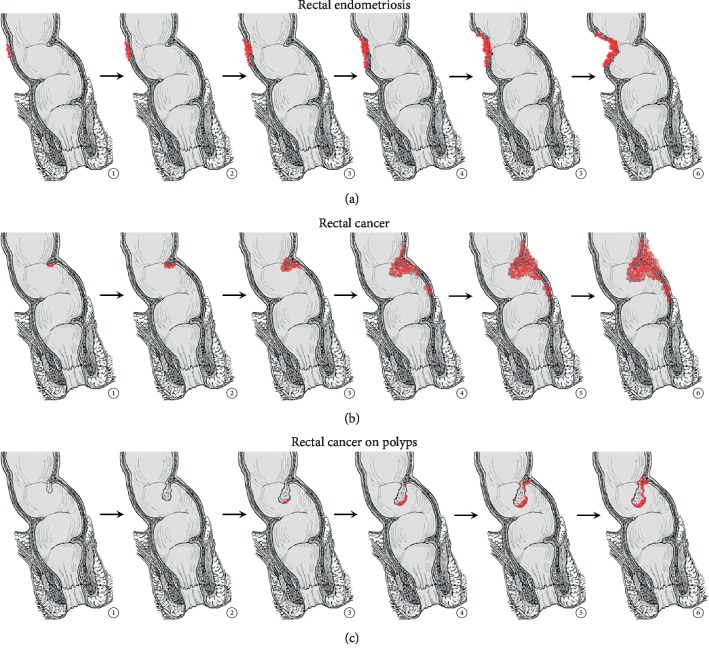
Differential growth of endometriosis and cancer of the rectum is of great help in the differential diagnosis.

Endometriosis is not cancer. Sonographically, it can be difficult to distinguish advanced rectal cancer from deep infiltrating endometriosis of the bowel although many signs have to be borne in mind to obtain a correct diagnosis. First of all, the patient's age may be the critical aspect as rectal cancer is found usually in advanced age although an increasing incidence in young patients aged younger than 40 years has been reported [30]. The second aspect is that endometriosis starts from the serosa and encroaches the bowel from outside while rectal cancer starts from the mucosa (or a polyp) before deepening through the muscularis layers (Figure 5 and [Supplementary-material supplementary-material-1]).

The third point is that large endometriosis nodules have a significant fibrosis component that contract the lesion and lead to a restriction of the lumen of the viscera (it resembles a “C” with the convex part towards the lumen) while a cancer lesion induces a lumen restriction because of its bulky mass. The fourth point is the clinical history of hematochezia that is unusual in endometriosis cases (unless these patients suffer from constipation and/or hemorrhoids) while it is a typical feature of bowel polyps and/or cancer.

Our report emphasizes that knowledge and awareness of the ultrasound features of endometriosis and other sonographically similar diseases can serve as an important mean to prevent physician misinterpretations of a condition that could have otherwise been properly treated. In fact, the major implications of missed diagnosis are a delay in the real diagnosis and subsequently in referring the patient to specialized centers. An appropriate diagnosis can facilitate an earliest intervention that could ultimately improve the clinical outcome.

## Figures and Tables

**Table 1:** Characteristics of all cases of Figure 1 at a glance.

Organ	Sonographic diagnosis			Differential diagnosis	
	Case number	US suspect	Appearance	US suspect	Differences
Rectum	#1, #2, #3, #4, #5, #6, #7, #8	Rectal endometriosis (confirmed diagnosis)	Lesions can present with different shapes, but they have always an anechoic appearance without posterior enhancement, can encroach the bowel lumen and their limits can be digitiform (#1), irregular (#2, #3), or smooth (#4, #5); very often the “sliding sign” is negative	Rectal polyp (see case #18 of Figure 4) Rectal diverticula (see cases #16 and #17 of Figure 3) Rectal cancer (see cases #19 and #20 of Figure 4)	(i) Age (ii) Family history (iii) Rectal bleeding (iv) Recta polyps and cancer grow from inside outwards while endometriosis starts from the serosa and encroaches the bowel (v) Only advanced rectal cancer can invade the serosa and pararectal tissue

**Table 2:** Characteristics of all cases of Figure 2 at a glance.

Organ	Sonographic diagnosis			Differential diagnosis	
	Case number	US suspect	Appearance	US suspect	Differences
Sigma and ileum	#9, #10, and #11	Multifocal endometriosis of the sigma-rectum (confirmed diagnosis)	Typical appearance of endometriosis of the bowel	Rectal polyp (see case #18 of Figure 4) Rectal cancer (see cases #19 and #20 of Figure 4)	(i) Age (ii) Family history (iii) Rectal bleeding (iv) Recta polyps and cancer can be both multifocal, but they grow from inside outwards while endometriosis starts from the serosa and encroaches the bowel
	#12	Endometriosis of the ileum (confirmed diagnosis)	The small bowel as seen in the vesico-uterine pouch and presented a typical endometriosis lesion (see cases #1 to #8)	Endometriosis of the vesico-uterine pouch	(i) Evidence of peristalsis (ii) Positive “sliding sign”

**Table 3:** Characteristics of all cases of Figure 3 at a glance.

Organ	Sonographic diagnosis			Differential diagnosis	
	Case number	US suspect	Appearance	US suspect	Differences
Bowel and pelvic diseases	#13	Tarlov cyst (confirmed diagnosis at MRI scan)	Anechoic cyst between the posterior wall of the rectum and the sacrum	Bowel endometriosis (see cases #1 to #8 of Figure 1)	(i) Endometriosis never affects the posterior wall of the bowel (ii) External layers of the rectal wall are intact and smooth
	#14	Presacral mass, probably a ganglioneuroma (confirmed diagnosis)	Apparently well-defined mass with fine low-level echoes; Doppler was considered not satisfactory	Bowel endometriosis (see cases #1 to #8 of Figure 1) Presacral endometriosis	(i) Endometriosis never affects the posterior wall of the bowel (ii) External layers of the rectal wall are intact and smooth (iii) Presacral endometriosis is never cystic as it appears as a solid not well-defined lesion
	#15	Appendicular abscess (confirmed diagnosis)	The caecum was dislocated down into the pelvis; the appendix was thick and attached to the ovary; an irregular hypoechoic area between the two organs was seen	Endometrioma	(i) High inflammatory blood tests (ii) No previous menstrual symptoms
	#16	Colonic diverticula (confirmed diagnosis by colonoscopy)	An external pouch of the sigmoid colon with a thickened wall and a hyperechoic content was seen	Bowel endometriosis (see cases #1 to #8 of Figure 1)	(i) It grows outwards (ii) Thick bowel wall (iii) Hyperechoic content
	#17	Colonic diverticular abscess (confirmed diagnosis)	An undefined oval mass was identified between the uterus and the sigmoid colon. A transverse section demonstrated the presence of a diverticulum (external pouch of the sigmoid colon with a thick wall and hyperechoic content) with an intense Doppler positivity	Endometrioma Corpus luteum Ovarian abscess Bowel endometriosis (see cases #1 to #8, Figure 1)	(i) Diverticula grow outwards (ii) Thick bowel wall (iii) Hyperechoic content (iv) Vascularization (v) Inflammatory blood tests

**Table 4:** Characteristics of all cases of Figure 4 at a glance.

Organ	Sonographic diagnosis			Differential diagnosis	
	Case number	US suspect	Appearance	US suspect	Differences
Colonic lesions	#18	Rectal polyp (confirmed diagnosis by endorectal ultrasound and colonoscopy)	A solid mass growing within the bowel lumen was seen with an intact serosa	Bowel endometriosis (see cases #1 to #8 of Figure 1) Rectal cancer (see cases #19 and #20 of Figure 4)	(i) Rectal bleeding (ii) Recta polyps and cancer grow from inside outwards while endometriosis starts from the serosa and encroaches the bowel
	#19 and #20	Rectal cancer (confirmed diagnosis)	A solid and irregular mass within the bowel lumen was seen; the serosa was intact	Bowel endometriosis (see cases #6 to #11 of Figures 1 and 2)	(i) Age (ii) Family history (iii) Endometriosis grows from the serosa (iv) Endometriosis, like cancer, can be multifocal

## Data Availability

The data that support the findings of this study are available on request from the corresponding author, MS. The data are not publicly available due to restrictions as they contain information that could compromise the privacy of research participants.
